# Olmesartan Combined With Amlodipine on Oxidative Stress Parameters in Type 2 Diabetics, Compared With Single Therapies: A Randomized, Controlled, Clinical Trial

**DOI:** 10.1097/MD.0000000000003084

**Published:** 2016-04-01

**Authors:** Giuseppe Derosa, Amedeo Mugellini, Rosa Maria Pesce, Angela D’Angelo, Pamela Maffioli

**Affiliations:** From the Department of Internal Medicine and Therapeutics, University of Pavia, and Fondazione IRCCS Policlinico San Matteo (GD, AM, RMP, ADA, PM); Center for the Study of Endocrine-Metabolic Pathophysiology and Clinical Research (GD); Laboratory of Molecular Medicine (GD, ADA); and PhD School in Experimental Medicine (PM), University of Pavia, Pavia, Italy.

## Abstract

To evaluate the effects of a fixed combination of olmesartan/amlodipine compared with olmesartan or amlodipine alone on some parameters of endothelial damage in diabetic, hypertensive patients.

We enrolled 221 patients; 74 were randomized to olmesartan 20 mg, 72 to amlodipine 10 mg, and 75 to olmesartan/amlodipine fixed combination 20/5 mg for 12 months. We assessed blood pressure monthly; in addition, we also assessed at baseline, and after 6 and 12 months, the following parameters: lipoprotein (a), myeloperoxidase (MPO), isoprostanes, and paraoxonase-1 (PON-1).

Blood pressure values obtained with fixed olmesartan/amlodipine combination were significantly lower than those reached with single monotherapies. There was a reduction of lipoprotein (a), and isoprostanes levels with olmesartan/amlodipine fixed combination, both compared with baseline, and with single monotherapies. On the other hand, there was an increase of PON-1 with fixed olmesartan/amlodipine combination, both compared with baseline, and with single drugs. All treatments reduced MPO compared with baseline; however, in group-to-group comparison, MPO reduction was greater with olmesartan/amlodipine fixed combination.

Fixed combination of olmesartan/amlodipine was more effective than single monotherapies in reducing oxidative stress, especially in increasing PON-1, and reducing isoprostanes levels in diabetic and hypertensive patients.

## INTRODUCTION

Oxidative stress and vascular inflammation are closely interrelated to endothelial dysfunction and vascular damage. Type 2 diabetes mellitus is characterized by a state of glycative and oxidative stress. Overproduction of the reactive oxygen species in diabetic patients may be due to chronic hyperglycemia, hyperinsulinemia, elevated free fatty acids (FFAs), and dyslipidemia, typical of this condition. Oxidative stress and mild chronic vascular inflammation also play a role in the pathophysiology of hypertension and atherosclerosis.^1^ In the literature, many studies reported that the increased oxidized low-density lipoprotein (LDL) in type 2 diabetes mellitus is correlated with an increased risk of cardiovascular complications.^2^ This increased susceptibility of LDL to oxidation is dependent on the antioxidant capacity of high-density lipoprotein (HDL)-associated paraoxonase-1 (PON-1). PON-1 is a glycoprotein expressed in several tissues, but it is mainly synthesized by the liver and circulates within HDL particles.^3^ The pleiotropic effects of some well-known antihypertensive agents and statins on oxidative stress and inflammation have been reported in the literature. Among the antihypertensive agents, olmesartan has been reported to protect against oxidative stress in rats, via the induction of nuclear factor-erythroid-2-related factor 2 (Nrf2) signaling pathways.^4^ Olmesartan medoxomil is a long-acting angiotensin II type I receptor (AT1R) antagonist approved for the treatment of mild to severe hypertension, alone or in combination with other agents. Olmesartan is therapeutically effective for the treatment of patients with heart failure by decreasing cytokines and oxidative stress through its anti-inflammatory effects.^5^ Regarding calcium channel blockers, in vitro studies proved that calcium channel blockers, including amlodipine, exhibit inhibitory effects on PON-1 at low concentrations.^6^ However, studies conducted in vivo in people with type 2 diabetes evaluating effects of antihypertensive agents on PON-1 are lacking. We already reported the effects of olmesartan/amlodipine combination on in hypertensive patients,^7^ but not in type 2 diabetic patients.

The aim of this study was to evaluate the effects of a fixed olmesartan/amlodipine combination 20/5 mg compared with olmesartan 20 mg or amlodipine 10 mg alone on some parameters indicative of endothelial damage and oxidative stress in patients with hypertension and type 2 diabetes mellitus. In particular, we were interested to evaluate if a fixed combination was better than single monotherapies in reducing blood pressure (BP), even at low dosage.

## METHODS

### Study Design

This randomized, double-blind, controlled study was conducted at the Department of Internal Medicine and Therapeutics, University of Pavia, and Fondazione IRCCS Policlinico San Matteo, Pavia, Italy.

The study protocol was conducted in accordance with the Declaration of Helsinki and its amendments, and the Good Clinical Practice Guidelines. It was approved by local Ethical Committee, and all patients provided written informed consent before entering the study (Trial registration: ClinicalTrials.gov NCT02064218).

### Patients

We enrolled 221 hypertensive patients with mild to moderate hypertension, type 2 diabetes mellitus, normocholesterolemic [low-density lipoprotein cholesterol (LDL-C) <160 mg/dL], overweight outpatients, and age ≥18 of either sex (Table 1).

**TABLE 1:**
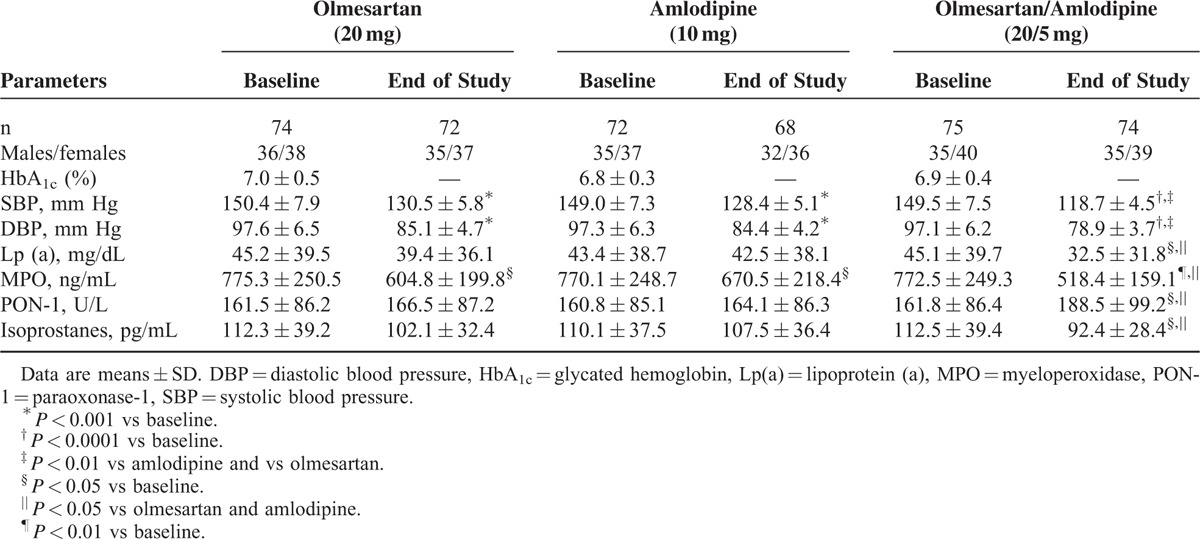
Data Change in the 3 Treatment Groups During the Study

Patients were evaluated for eligibility according to the following inclusion criteria: systolic BP (SBP) ≥ 140 mm Hg < 180 mm Hg and/or diastolic BP (DBP) ≥ 90 mm Hg < 105 mm Hg; and well-controlled type 2 diabetes mellitus [glycated hemoglobin (HbA_1c_) ≤7.5%].

The exclusion criteria were secondary hypertension; severe hypertension (SBP ≥180 mm Hg or DBP ≥105 mm Hg); hypertrophic cardiomyopathies due to etiologies other than hypertension, history of heart failure, history of angina, stroke, transient ischemic cerebral attack, coronary artery bypass surgery, or myocardial infarction any time before visit 1; concurrent known symptomatic arrhythmia; liver dysfunction (aspartate aminotransferase or alanine aminotransferase values exceeding 2-fold the upper limit); creatinine >1.5 mg/dL; and known hypersensitivity to the study drugs. Pregnant women and women of childbearing potential were excluded. Suitable subjects, identified from review of case notes and/or computerized clinic registers, were contacted personally or by telephone.

### Treatments

Patients fulfilling the inclusion criteria and not satisfying the exclusion criteria were randomized to amlodipine 10 mg/day, or olmesartan 20 mg/day, or to a fixed combination of olmesartan/amlodipine 20/5 mg/day for 12 months. Olmesartan, amlodipine, and olmesartan/amlodipine were supplied as identical, opaque, white capsules in coded bottles to ensure the blind status of the study. Randomization was done using a drawing of envelopes containing randomization codes prepared by a statistician. A copy of the code was provided only to the responsible person performing the statistical analysis. The code was only broken after database lock, but could have been broken for individual subjects in cases of an emergency. Medication compliance was assessed by counting the number of pills returned at the time of specified clinic visits. At baseline, we weighed participants and gave them a bottle containing a supply of the study medication for at least 100 days. Throughout the study, we instructed patients to take their first dose of new medication on the day after they were given the study medication. At the same time, all unused medication was retrieved for inventory. All medications were provided free of charge.

### Diet and Exercise

Patients were already following a controlled-energy diet (near 600 kcal daily deficit) based on American Heart Association (AHA) recommendations that included 50% of calories from carbohydrates, 30% from fat (6% saturated), and 20% from proteins, with a maximum cholesterol content of 300 mg/day and 35 g/day of fiber. Patients were not treated with vitamins or mineral preparations during the study.^8^

Standard diet advice was given by a dietitian and/or specialist doctor. The dietitian and/or specialist doctor periodically provided instruction on dietary intake recording procedures as part of a behavior modification program and then later used the subject's food diaries for counseling. Individuals were also encouraged to increase their physical activity by walking briskly for 20 to 30 minutes, 3 to 5 times/week, or by cycling. The recommended changes in physical activity throughout the study were not assessed.

### Assessments

Before starting the study, all patients underwent an initial screening assessment that included a medical history, physical examination, vital signs, and a 12-lead electrocardiogram. We assessed BP every month; in addition, we also collected blood sample to assess at baseline, and after 6 and 12 months, the following parameters: lipoprotein (a) [Lp(a)], myeloperoxidase (MPO), isoprostanes, and PON-1.

All plasmatic parameters were determined after a 12-hour overnight fast. Venous blood samples were taken from all patients between 08.00 and 09.00 a.m. We used plasma obtained by addition of Ethylene Diamine Tetraacetic Acid Disodium (1 mg/mL), and centrifuged at 3000*g* for 15 minutes at 4°C. Immediately after centrifugation, the plasma samples were frozen and stored at −80°C for no more than 3 months. All measurements were performed in a central laboratory.

Blood pressure measurements were obtained from each patient (left arm) in the sitting position by physicians blinded to treatment using a standard mercury sphygmomanometer (Erkameter 3000; ERKA, Bad Tolz, Germany) (Korotkoff I and V), with a cuff of appropriate size. BP has been always measured in the morning before daily drug intake (ie, at trough 22–24 hours after dosing) and after the subject has rested 10 minutes in a quiet room. Three successive BP readings were obtained at 1-minute intervals and averaged.

Heart rate was measured by pulse palpation for 30 seconds, just before the BP measurements.

Glycated hemoglobin level was measured by a high-performance liquid chromatography method (DIAMAT, Bio-Rad; normal values 4.2%–6.2%), with intra and interassay coefficients of variation (CsV) of <2%.^9^

Plasma glucose was assayed by glucose-oxidase method (GOD/PAP, Roche Diagnostics, Mannheim, Germany) with intra and interassay CsV of <2%.^10^

Lipoprotein(a) was measured by a sandwich enzyme-linked immunosorbent assay (ELISA) method, which is insensitive to the presence of plasminogen, using the commercial kit Macra-Lp(a) (SDI, Newark, DE); the intra and interassay CsV of this method were 5% and 9%, respectively.^11,12^

Myeloperoxidase was assessed using commercially available ELISA kits according to manufacturer's instructions (R&D Systems, Minneapolis, MN). The intra and interassay CsV were 7.7% and 8.3%, respectively.^13^

The level of isoprostanes in serum was determined by commercially available ELISA kit (Cayman Chemicals, Ann Arbor, MI).^14^

Paraoxonase-1 activity in serum was measured using paraoxon as a substrate in the presence of 2 mM Ca^+2^ in 100 mM Tris-HCL buffer (pH = 8.0).^15^

### Statistical Analysis

Data are expressed as mean ± standard deviation (SD). The statistical analysis of the data was performed by the statistical analysis software (SAS) system, version 6.12 (SAS Institute, Inc., Cary, NC). The differences between the 2 groups in baseline characteristics were analyzed by the 2-tailed Student *t* test. Comparisons within and between groups were assessed by a 2-way analysis of variance (ANOVA) for repeated measurements. Differences between baseline and after 12 months’ treatment in each group in BP and oxidative stress parameters were analyzed with the Wilcoxon signed-rank test. Comparisons of changes in BP and oxidative stress parameters between the 2 groups were performed with the Mann–Whitney *U* test.^16^ Findings of *P* <0.05 were considered significant. Considering as clinically significant a difference of at least 10% compared with the baseline and an alpha error of 0.05, the actual sample size was adequate to obtain a power higher than 0.80 for all measured variables.

## RESULTS

### Study Sample

**FIGURE 1:**
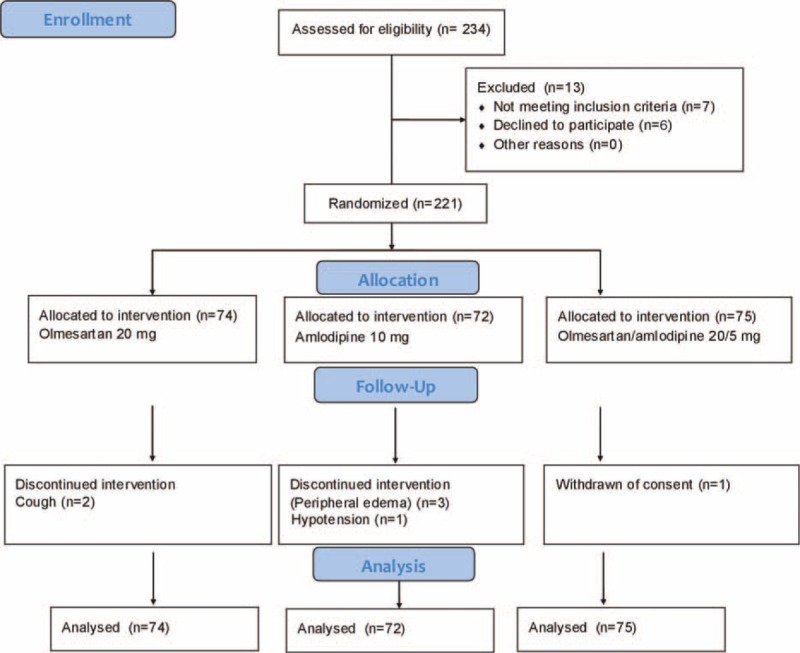
CONSORT 2010 flow diagram.

We enrolled 221 patients; 74 were randomized to olmesartan 20 mg, 72 to amlodipine 10 mg, and 75 to olmesartan/amlodipine fixed combination 20/5 mg. In all, 214 patients completed the study. Seven patients did not complete the study and the reasons for prematurely withdrawal included: peripheral edema (3 patients), cough (2 patients), hypotension (1 patient), and withdraw of consent (1 patient) (Figure 1).

### Blood Pressure

Blood pressure decreased in all groups compared with baseline (*P* < 0.001 vs baseline with olmesartan and amlodipine monotherapies, and *P* < 0.0001 vs baseline with fixed olmesartan/amlodipine combination). BP values obtained with fixed combination were significantly lower than those reached with single monotherapies (*P* < 0.01 for both) (Table 1).

### Oxidative Stress Parameters

There was a reduction of Lp(a), and isoprostanes levels with olmesartan/amlodipine fixed combination, both compared with baseline (*P* < 0.05), and to single monotherapies (*P* < 0.05). On the other hand, there was an increase of PON-1 with fixed olmesartan/amlodipine combination both compared with baseline (*P* < 0.05), and to single drugs (*P* < 0.05 for both), but not with single monotherapies. All treatments reduced MPO compared with baseline (*P* < 0.05 for olmesartan, *P* < 0.05 for amlodipine, and *P* < 0.01 for fixed olmesartan/amlodipine combination); however, in group-to-group comparison, MPO reduction was greater with olmesartan/amlodipine fixed combination (Table 1).

## DISCUSSION

In our study, we observed that a fixed olmesartan/amlodipine combination better improved oxidative stress, increasing PON-1 levels and reducing isoprostanes levels. Our results are in line with what was already reported in the OLAS (OLmesartan/Amlodipine vs olmesartan/hydrochlorothiazide in metabolic Syndrome), trial, which suggested that combination therapy comprising an angiotensin type 1 receptor blocker plus a calcium channel blocker may offer advantages in patients at high cardiovascular risk and with underlying metabolic issues.^17^ We also recorded a better effect of the fixed combination in reducing LP(a) and MPO levels that has been recognized as new emerging markers of cardiovascular risk.^18^ In particular, Lp(a) is capable of deleteriously altering the balance between the procoagulant and anticoagulant, proinflammatory and anti-inflammatory, and vasorelaxing and vasoconstricting properties of the endothelium. Lp(a) has been reported to potentiate thrombosis, inhibiting the binding of plasminogenic binding proteins on the surface of endothelial cells, thus inhibiting the conversion of plasminogen to plasmin and hence fibrinogen and fibrin degradation.^19^ For this reason, Lp(a) could play an important role in essential hypertension pathogenesis and could be considered as an individual risk factor in hypertensive patients.^20^ On the other hand, MPO reduces nitric oxide (NO) bioavailability by direct consumption of NO and production of reactive oxygen species that oxidize tetrahydrobiopterin to its inactive form, which, in turn, uncouples endothelial NO synthase.^21,22^ Regarding the reasons why single monotherapies did not improve studied parameters, whereas fixed combination did, this is probably due to a synergic effects of the 2 antihypertensive agents taken together.

As far as our knowledge is concerned, our study is the first to directly compare the effects of a fixed olmesartan/amlodipine combination on oxidative stress markers in diabetic patients.

Of course, our study has some limitations such as the short study duration; moreover, we assessed only some oxidative stress markers, focusing our attention on a few of them. In addition, we chose different therapies at different dosages (maximum dosage of amlodipine, intermediate dosage of olmesartan, and combination), obtaining different antihypertensive effects and different systolic and diastolic reductions. It cannot be excluded that all the observed changes in the selected parameters of endothelial dysfunction might be related to BP reductions rather than to the intrinsic properties of the drugs.

## CONCLUSIONS

Fixed combination of olmesartan/amlodipine was more effective than single monotherapies in reducing oxidative stress, especially in increasing PON-1 and reducing Lp(a) and isoprostanes levels in diabetic and hypertensive patients.
